# The Gut Microbiota: A Novel Player in Autoimmune Hepatitis

**DOI:** 10.3389/fcimb.2022.947382

**Published:** 2022-07-11

**Authors:** Zilu Cheng, Ling Yang, Huikuan Chu

**Affiliations:** Division of Gastroenterology, Union Hospital, Tongji Medical College, Huazhong University of Science and Technology, Wuhan, China

**Keywords:** intestinal microbiota, autoimmune hepatitis, gut-liver axis, probiotics, fecal microbiota transplantation

## Abstract

Autoimmune hepatitis (AIH) is a chronic immune-mediated liver disease distributed globally in all ethnicities with increasing prevalence. If left untreated, the disease will lead to cirrhosis, liver failure, or death. The intestinal microbiota is a complex ecosystem located in the human intestine, which extensively affects the human physiological and pathological processes. With more and more in-depth understandings of intestinal microbiota, a substantial body of studies have verified that the intestinal microbiota plays a crucial role in a variety of digestive system diseases, including alcohol-associated liver disease (ALD) and non-alcoholic fatty liver disease (NAFLD). However, only a few studies have paid attention to evaluate the relationship between AIH and the intestinal microbiota. While AIH pathogenesis is not fully elucidated yet, some studies have indicated that intestinal microbiota putatively made significant contributions to the occurrence and the development of AIH by triggering several specific signaling pathways, altering the metabolism of intestinal microbiota, as well as modulating the immune response in the intestine and liver. By collecting the latest related literatures, this review summarized the increasing trend of the aerobic bacteria abundance in both AIH patients and AIH mice models. Moreover, the combination of specific bacteria species was found distinct to AIH patients, which could be a promising tool for diagnosing AIH. In addition, there were alterations of luminal metabolites and immune responses, including decreased short-chain fatty acids (SCFAs), increased pathogen associated molecular patterns (PAMPs), imbalanced regulatory T (Treg)/Th17 cells, follicular regulatory T (TFR)/follicular helper T (TFH) cells, and activated natural killer T (NKT) cells. These alterations participate in the onset and the progression of AIH *via* multiple mechanisms. Therefore, some therapeutic methods based on restoration of intestinal microbiota composition, including probiotics and fecal microbiota transplantation (FMT), as well as targeted intestinal microbiota-associated signaling pathways, confer novel insights into the treatment for AIH patients.

## Introduction

Autoimmune hepatitis (AIH) is a chronic progressive immune-mediated liver disease. It is predominantly characterized by the presence of autoantibodies, elevated levels of serum transaminase and immunoglobulin G, and liver histologic interface hepatitis (Cowling et al., 1956; Manns et al., 2010). AIH affects the health state of people in all age groups around the globe, with a female propensity (Cowling et al., 1956). The incidence of AIH is estimated at 4/100,000-24.5/100,000 per year in the Asia-pacific region, and it also increases by years in China (Lee et al., 2001; Jalihal et al., 2009; Haider et al., 2010; Ngu et al., 2010; Delgado et al., 2013; European Association for the Study of the Liver, 2015; Kim et al., 2017). AIH is a serious immune-mediated liver disease, which will lead to some detrimental consequences, including cirrhosis and liver failure (European Association for the Study of the Liver, 2015). Up to date, AIH pathogenesis is not completely elucidated. Genetic predisposition, environmental factors and immune tolerance breakdown are identified as significant contributors to the occurrence and the development of AIH (Wei et al., 2020). Notably, some studies have reported that intestinal microbiota dysbiosis had an intimate association with AIH (Yuksel et al., 2015; Liwinski et al., 2020; Wei et al., 2020).

The intestinal microbiota inhabits the human gut tract, mainly comprised of more than 100 trillion bacteria, and their genomes contain 150-fold more genes than humans (Qin et al., 2010).The intestinal microbiota is capable of coexisting with the host harmoniously and exerting significant influence on its pathological and physiological processes, such as assisting digestion and absorption of nutrients, preventing the colonization of pathobiont, and maintaining a steady immune system (Nicholson et al., 2012). A substantial body of studies have suggested that intestinal microbiota dysbiosis plays a significant role in immune-mediated disorders (Liwinski et al., 2020; Wang et al., 2021a), including AIH. It is commonly accepted that intestinal barrier destruction, intestinal microbiota translocation, as well as immune homeostasis breakdown contribute to the onset and progression of AIH (Yuksel et al., 2015; Wei et al., 2020; Yang et al., 2021).

Currently, the therapeutic methods for AIH are glucocorticoid or a combination with azathioprine (Mack et al., 2020). However, glucocorticoid and azathioprine have many negative effects, such as central obesity, osteoporosis, myelosuppression, and liver function damage (Lee et al., 2014; Mack et al., 2020). The therapeutic needs of patients not tolerating standard management or not achieving remission remain unmet (Mack et al., 2020). A number of researches associated with AIH animal models and AIH patients highlight the importance of “intestinal liver crosstalk” in AIH pathogenesis (Liang et al., 2021; Wang et al., 2021b), which offers a promise of novel diagnostic and therapeutic methods. Therefore, great significance should be attached to deeply explore the specific impact of intestinal microbiota on AIH and its associated mechanisms, and further discuss the efficacy and safety of several potential therapies, including probiotics, fecal microbiota transplantation (FMT), as well as some pharmacological agents which target intestinal microbiota-associated signaling pathways.

## The Gut-Liver Axis

The theory of the “the gut-liver axis” was initially raised by Marshall (Volta et al., 1987), which refers to an intimate anatomical, functional, and bidirectional interaction of the gut and the liver, predominantly *via* the portal circulation (Abdel-Misih and Bloomston, 2010). The gut-liver axis is identified as a pivotal contributor to the occurrence and development of multiple liver disease (Saffouri et al., 2019; Scorletti et al., 2020) and autoimmune disease (Allegretti et al., 2019; Fretheim et al., 2020; Wang et al., 2021a).

In healthy conditions, the intestinal epithelium constitutes a natural barrier to confer adequate protection against the intestinal microbiota as well as their metabolites through a tight junction, antibacterial molecules and mucus layer (Beyaz Coşkun and Sağdiçoğlu Celep, 2021). The liver is widely thought to be the first organ exposed to gut-derived harmful substances, including bacteria and bacterial metabolites (Seki and Brenner, 2008), but only a small quantity of them can move to the liver, where they are detoxified or diminished by the immune system in a healthy status (Lin et al., 2015). Microbiota dysbiosis leads to the destruction of intestinal barrier (Lin et al., 2015), which further results in the translocation of intestinal microbiota from the gut to the liver. The excessive gut-derived microbial toxins may destroy liver homeostasis by aberrantly activating the innate immune system and triggering signaling pathways related to liver inflammatory responses (Pradere et al., 2010). With compromised intestinal barrier and disrupted immune homeostasis, the intestinal microbiota, which can be regarded as a continuous source of antigens, initiates, maintains, and perpetuates the autoimmune responses in AIH (Sánchez et al., 2015; Van Praet et al., 2015; Ignacio et al., 2016).

## The Relationship Between AIH and Intestinal Microbiota

Intestinal microbiota makes great contributions to the onset and progression of AIH (Yuksel et al., 2015; Liwinski et al., 2020; Wei et al., 2020). Germ-free mice had a fair resistance to fulminant hepatitis induced by concanavalin A (Con A), which contrasted sharply with specific pathogen free mice (Wei et al., 2016). Moreover, gentamycin mitigated the liver injury induced by Con A through depleting gut-derived gram-negative bacteria, concomitantly with reduced liver immune cells infiltration, whereas administration of exogenous pathogenic bacteria aggravated Con A-induced acute hepatitis (Chen et al., 2014). Furthermore, in a recent study, compared to the controls, the antibiotic-treated mice exhibited AIH phenotypes after being transplanted with fecal microbiota from mice exposed to trichloroethene (TCE), accompanied by increased systematic autoantibodies and aggravated hepatic inflammation (Wang et al., 2021a). The afore-mentioned evidence supported an intimate linkage between the etiology of AIH and intestinal microbiota.

Besides, intestinal microbiota has changed significantly in AIH patients and animal models compared to the healthy group (**Table 1**) (Yuksel et al., 2015; Elsherbiny et al., 2020; Lou et al., 2020; Wei et al., 2020). Overall, the biodiversity of the intestinal microbiome has decreased remarkably, and the relative abundance of aerobic or facultative anaerobic bacteria increased (Lin et al., 2015; Yuksel et al., 2015; Elsherbiny et al., 2020; Wei et al., 2020). The taxonomic analysis of fecal microbiome from the controls as well as AIH patients showed that at the phylum level, Verrucomicrobia abundance remarkably increased while Synergistetes and Lentisphaerae abundance remarkably decreased in patients with AIH compared to healthy communities (Elsherbiny et al., 2020; Lou et al., 2020). Of note, Synergistetes and Lentisphaerae belong to anaerobic bacteria (Limam et al., 2010; Aoyagi et al., 2020), and Synergistetes have the capacity to participate in the anaerobic dissimilation of acetate (Aoyagi et al., 2020). However, the changes in Bacteroidetes, Firmicutes and Proteobacteria were controversial in different studies (Elsherbiny et al., 2020; Wei et al., 2020). At the genus level, compared to healthy group, *Veillonella*, *Streptococcus, Klebsiella, Akkermansia, Blautia, Eubacterium, Butyricicoccus* and *Haemophilus* were mainly enriched in AIH patients while *Bifidobacterium, Ruminococcus, Clostridiales, Rikenellaceae, Oscillospira, Sutterella, Parabacteriods* and *Coprococcus* were retracted in such patients (Lin et al., 2015; Elsherbiny et al., 2020; Liwinski et al., 2020; Lou et al., 2020; Wei et al., 2020). Furthermore, there were different outcomes concerning the abundance of *Lactobacillus, Faecali bacterium* and *Lachospiraceae* (Liwinski et al., 2020; Lou et al., 2020; Wei et al., 2020). Some researches established AIH mouse model that virtually mimicked the condition of AIH patients, and analyzed the fecal microbiome of these models. The results suggested that at the phylum level, compared to the controls, Proteobacteria and Bacteroidetes abundance were increased, and the increment of Proteobacteria (facultative anaerobic bacteria) was thought to correlate with inflammation, epithelial dysfunction, as well as the breakdown of host-microbiota homeostasis (Litvak et al., 2017). At the genus level, compared to healthy community, *Akkermansiaceae* and *Lachospiraceae* abundance were increased while *Lactobacillus, Bifidobacterium* and *Rikenellaceae* abundance were decreased (Yuksel et al., 2015; Wang et al., 2021a). Besides the afore-mentioned alterations of intestinal microbiota from fecal samples, *Enterococcus gallinarum* was remarkably enriched in the liver of AIH patients (Manfredo Vieira et al., 2018). The combination of *Lactobacillus, Veillonella, Clostridiales* and *Oscillospira* was regarded as a potent biomarker to make a distinction between healthy individuals and AIH patients with an area under curve (AUC) value of 78% (Wei et al., 2020), and another five genera including *Veillonella, Lachnospiraceae, Roseburia*, *Ruminococcaceae* and *Bacteroides* were able to discriminate AIH patients from healthy individuals, which were confirmed to achieve an AUC of 83.25% (Lou et al., 2020). These results suggested that the specific alterations of intestinal microbiota could be used as potent biomarkers to distinguish AIH patients from healthy communities.

**Table 1:** Changes of intestinal microbiota associated with AIH in feces.

Participants	Comparison	Change of intestinal microbiota		Method	Ref
		Increased	Decreased		
AIH patients (n=24) Healthy individuals (n=8)	AIH vs Healthy		*Bifidobacterium; Lactobacillus*	16S rDNA quantitative PCR	Lin et al. (2015)
HLA-DR3 NOD mice WT NOD mice	AIH vs Healthy	Proteobacteria; Bacteriodetes		16S rRNA sequencing	Yuksel et al. (2015)
AIH patients (n=72) Healthy individuals (n=95)	AIH vs Healthy	*Streptococcus; Veillonella; Lactobacillus*	*Faecalibacterium;* *Bifidobacterium*	16S rRNA sequencing	Liwinski et al. (2020)
AIH patients (n=37) Healthy individuals (n=78)	AIH vs Healthy	Verrucomicrobia; *Veillonella;* *Faecalibacterium;* *Akkermansia*	Lentisphaerae; Synergistetes; *Pseudobutyrivibrio;* *Lachnospira;* *Ruminococcaceae*	16S rRNA sequencing	Lou et al. (2020)
AIH patients (n=15) Healthy individuals (n=10)	AIH vs Healthy	Firmicutes; Bacteroides; Proteobacteria; *Faecalibacterium; Blautia; Streptococcus;* *Haemophilus; Bacteroides; Veillonella; Eubacterium; Lachnospiraceae;* *Butyricicoccus*	*Prevotella; Parabacteroides; Dilaster*	16S rRNA sequencing	Elsherbiny et al. (2020)
AIH patients (n=91) Healthy individuals (n=98)	AIH vs Healthy	*Veillonella;* *Klebsiella;* *Streptococcus; Lactobacillus*	*Clostridiales; RF39;* *Ruminococcaceae; Rikenellaceae; Oscillospira; Parabacteroides;* *Coprococcus*	16S rRNA sequencing	Wei et al.^10^
TCE-treated mice Control mice	AIH vs Healthy	*Akkermansiaceae;Lachnospiraceae*	*Lactobacillaceae; Rikenellaceae; Bifidobacteriaceae*	16S rRNA sequencing	Wang et al^15^

Comparison of condition A vs condition B: ↑signifies an increase in condition A relative to condition B. ↓signifies a decrease in condition A relative to condition B.

AIH, autoimmune hepatitis; HLA, human leukocyte antigen; NOD, nonobese-diabetic; WT, wild type; TCE, Trichloroethene.

Some specific microbiome is also confirmed to correlate with the severity of AIH (Lin et al., 2015), such as *Veillonella*, as it exhibits the strongest relativity to AIH (Wei et al., 2020). The abundance of *Veillonella* shows a positive correlation with the level of serum aspartate aminotransferase (AST), as well as the inflammation grades of the liver (Wei et al., 2020). A decline of *Bifidobacterium* is also related to the increased disease activity and failure to achieve remission (Liwinski et al., 2020). Moreover, the increment of plasma lipopolysaccharide (LPS) induced by dysbiosis in AIH is confirmed to correlate with advanced stages of the disease (Lin et al., 2015). These biomarkers can be used as noninvasive hallmarks to assist the diagnosis of AIH as well as the evaluation of the disease severity, which needs rigorous evaluation and further investigation.

Furthermore, with the increment of researches associated with non-bacterial communities in the gut, including fungi, viruses, and archaea, a number of literatures have reported the alterations of fungi, viruses, and archaea in some chronic liver diseases, including alcohol-associated liver disease (ALD) (Yang et al., 2017; Lang et al., 2020; Hartmann et al., 2021) and non-alcohol fatty liver disease (NAFLD) (You et al., 2021). It appears plausible to speculate that these non-bacterial communities also contribute to the progression of AIH despite the lack of reported relevant literatures, which warrants the emergence of new evidence.

In summary, intestinal microbiota in AIH patients and animal models has changed remarkably, with a decreased biodiversity and a conversion to aerobic or facultative anaerobic microorganisms (Lin et al., 2015; Yuksel et al., 2015; Elsherbiny et al., 2020; Wei et al., 2020). The specific alterations of the intestinal microbiota are conducive to making a distinction between AIH patients and healthy individuals (Lou et al., 2020; Wei et al., 2020). Moreover, some species of intestinal microbiota, such as *Veillonella* and *Bifidobacterium*, as well as bacterial products like LPS, are closely related to the disease severity (Lin et al., 2015; Liwinski et al., 2020; Wei et al., 2020), which are likely to be adjuvant to evaluate the progression of AIH. With a more in-depth understanding of non-bacterial communities, it is reasonable to speculate that they putatively take part in the progression of AIH, which warrants the emergence of new evidence.

## The Influential Mechanisms of Altered Intestinal Microbiota in AIH

### Metabolite Pathway

The alterations of the intestinal microbiota in AIH disease model exert impact on the metabolism of luminal contents, including short-chain fatty acid (SCFA) (Liwinski et al., 2020; Lou et al., 2020), amino acid (Elsherbiny et al., 2020; Wei et al., 2020) as well as bile acid (Kayama et al., 2020; Wei et al., 2020), which affect the integrity and permeability of intestinal barrier and immune homeostasis.

SCFAs consist of acetic acids, propionic acids, and butyric acids, which belong to organic acids produced from undigested dietary fibers fermentation by intestinal bacteria (Bergman, 1990; Ríos-Covián et al., 2016; Martin-Gallausiaux et al., 2021). Their quantity and relative abundance are regarded as one of the biomarkers of health status (Ríos-Covián et al., 2016; Blaak et al., 2020). In the AIH disease model, the decrease of anaerobic bacteria, such as *Ruminococcus* (Lou et al., 2020), leads to the decrease of SCFAs (Liwinski et al., 2020; Lou et al., 2020), which exacerbates the inflammation response in AIH (Lou et al., 2020). Moreover, the administration of *Bifidobacterium* animal lactic acid 420 (B420) in experimental autoimmune hepatitis (EAH) mice increased the abundance of *Clostridium*, which had a correlation with the production of SCFAs, mitigating autoimmune hepatitis and intestinal barrier injury (Zhang et al., 2020). Therefore, it appears plausible that the altered intestinal microbiota in AIH causes the decrease of SCFAs, exacerbating the disease’s progression. Putative mechanisms are described as follows. First of all, the production of SCFAs is accompanied by the decrease of luminal pH, which is not conducive to the growth of intestinal pathobiont, thus contributing to the restoration of altered intestinal microbiota in AIH (Perman et al., 1981). Moreover, several studies have shown that the administration of SCFAs alleviated inflammatory responses of systematic autoimmune diseases mediated by lymphocytes through increasing Tregs cells and reducing Th1 cells (Mizuno et al., 2017), which indicated that SCFAs might be able to mitigate the inflammatory injury in AIH. Furthermore, butyric acids, as the most significant constituent of SCFAs, can stimulate intestinal epithelial cells (IECs) so as to induce mucin expression, resulting in the alteration of bacterial adhesion (Jung et al., 2015) and the improvement of the integrity of tight junction (Peng et al., 2009). In the meanwhile, the pretreatment of butyric acid can reduce the elevated level of proinflammatory factors induced by LPS, including tumor necrosis factor-α (TNF-α), interleukin (IL)-1β and IL-6, which also can stimulate anti-inflammatory factor secretion, like IL-10 (Wang et al., 2017). Therefore, it seems that the decrease of SCFAs is associated with intestinal barrier destruction and immune homeostasis breakdown, thus exacerbating the progression of AIH.

The altered arginine metabolism in AIH induced by the intestinal microbiota could decrease the serum polyamine level (Tabor and Tabor, 1985; Wei et al., 2020). Such a decrease is unfavorable for the differentiation and maturation of intestinal resident immune cells (Löser et al., 1999), thereby impacting the intestinal immune responses in AIH patients. Besides, the increase of branched-chain amino acids in AIH patients (Elsherbiny et al., 2020), including Leucine, Valine as well as Isoleucine, are conducive to upregulating innate and adaptive immune responses and modulating intestinal barrier function *via* multiple key signaling pathways (Negro et al., 2008; Nakamura, 2014)., thus participating in the development of the disease.

The secondary bile acid is thought to be a ligand for G-protein coupled bile acid receptor 1(GPBAR1) expressed on natural killer T (NKT) cell (Maruyama et al., 2002; Kawamata et al., 2003). The decreased abundance of *Clostridium* in AIH brings about the decrease of secondary bile acid (Kayama et al., 2020), which inhibits the polarization of NKT 10 cells from NKT cells and the secretion of anti-inflammatory cytokines IL-10 *via* the inactivation of GPBAR1, thereby alleviating liver injury in Con A-induced hepatitis (Biagioli et al., 2019).

In summary, dysbiosis in AIH disease models exerted influence on the metabolism of intestinal microbiota, and with it the altered concentrations of various intestinal metabolites, including the decrease of SCFAs, polyamine, and secondary bile acids, and the increase of branched-chain amino acids (Elsherbiny et al., 2020; Kayama et al., 2020; Liwinski et al., 2020; Lou et al., 2020; Wei et al., 2020). These alterations are identified as great contributors to intestinal barrier destruction, immune homeostasis breakdown, and inflammatory injury aggravation, thus giving impetus to the progression of AIH.

### Receptor Pathway

**Figure 1:**
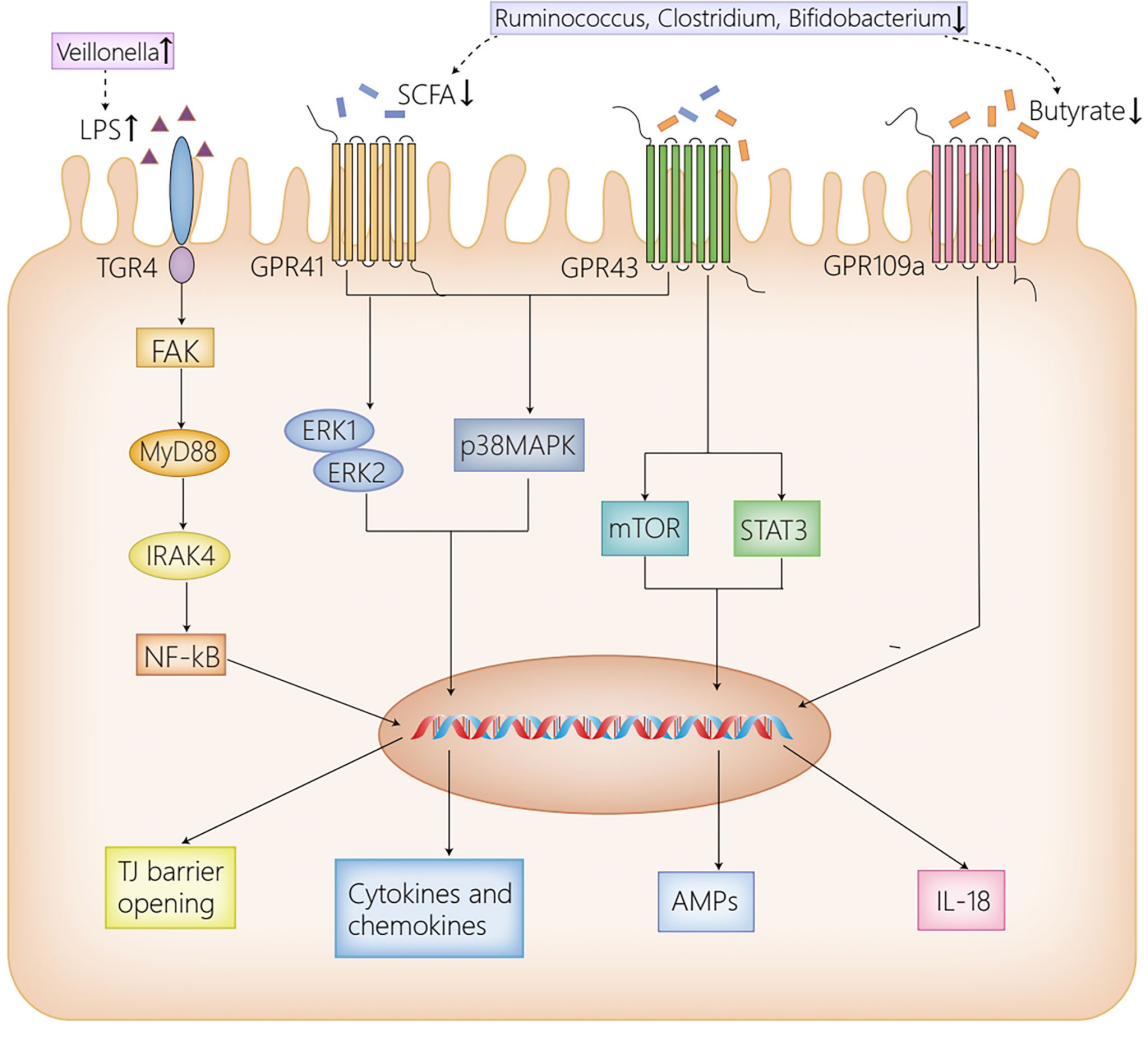
The receptor pathways in the intestine. High dose LPS induced by the increased abundance of *Veillonella* in AIH activates TLR4 expressed on IECs, then leads to the phosphorylation and activation of FAK, which regulates the activation of MyD88 and IRAK4, ultimately disrupting the intestinal TJ barrier. GPR41/43 on IECs activates ERK1/2 and p38MPAK signaling pathways by SCFAs, contributing to cytokines and chemokines secretion, which mediate protective immunity. Moreover, the activation of GPR43 by butyrate induces AMPs production by activating mTOR and STAT3. Furthermore, GPR109a promotes the expression of IL-18, which confers protection against intestinal inflammation. Herein, the reduction of *Ruminococcus, Clostridium* and *Bifidobacterium* in AIH, which results in the decrease of SCFAs, inhibits the afore-mentioned signaling pathways activated by SCFAs, thus contributing to intestinal barrier disruption. AIH, autoimmune hepatitis; IECs, intestinal epithelial cells; LPS, lipopolysaccharide; TLR4, Toll-like receptor 4; FAK, focal adhesion kinase; MyD88, myeloid differentiation factor 88; IRAK4, IL-1R–associated kinase 4; NF-kB, nuclear factor kappa B; TJ, tight junction; SCFAs, short-chain fatty acids; GPR41/43/109a, G-protein-coupled receptors 41/43/109a; ERK1/2, extracellular signal-related kinase 1/2; MAPK, mitogen-activated protein kinase; mTOR, mammalian target of rapamycin; STAT3, signal transducers and activator of transcription 3; AMPs, antimicrobial peptides; IL-18, interleukin 18.

**Figure 2:**
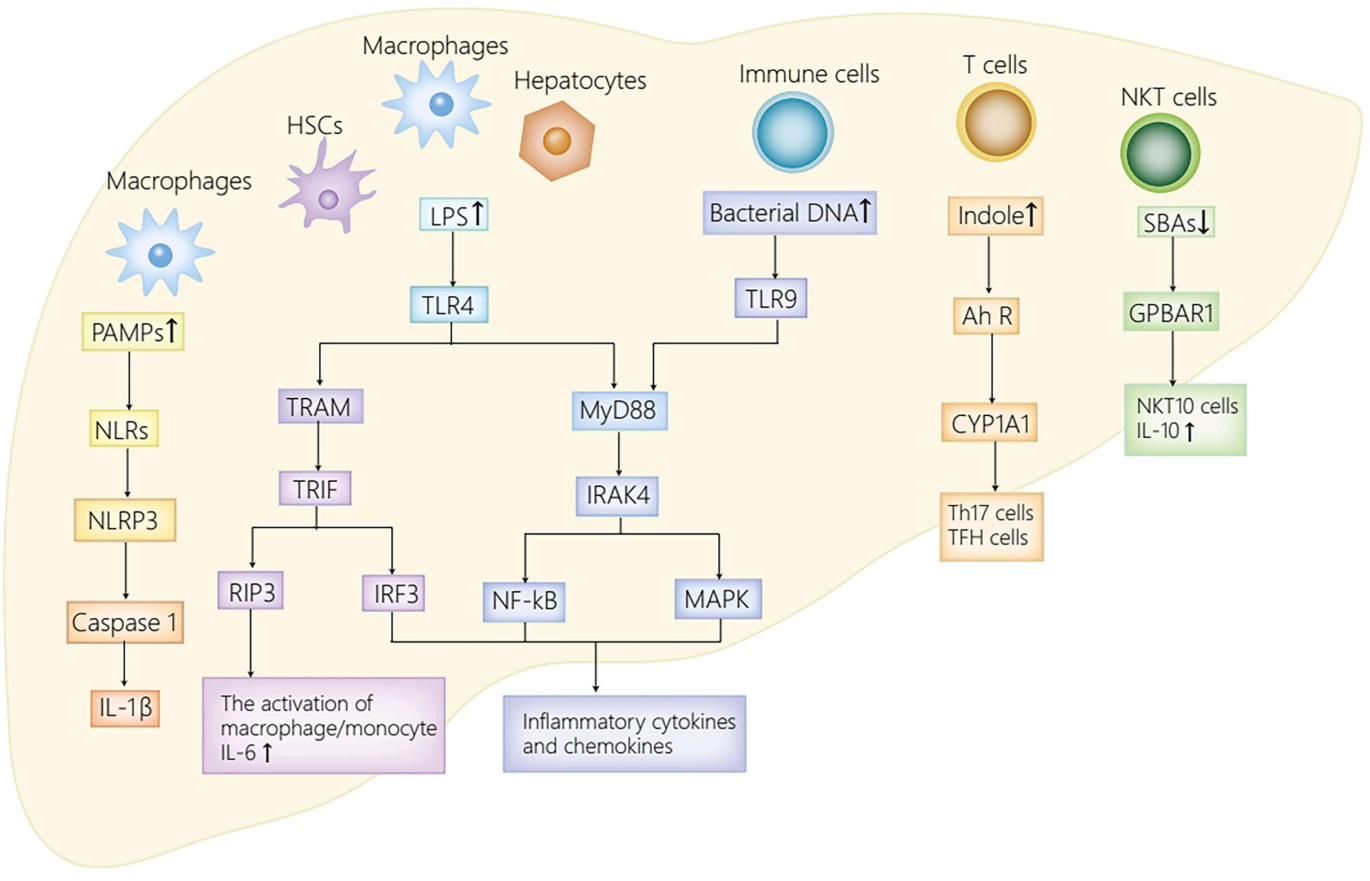
The receptor pathways in the liver. The activation of NLRs by PAMPs activates NLRP3 inflammasome, thus promoting caspase-1 cleavage as well as IL-1β secretion. TLR4 expressed on HSCs, Kupffer cells and hepatocytes enhances inflammatory chemokines and cytokines secretion by activating IRF3, MAPK and NF-kB. Moreover, LPS also stimulates TLR4 on macrophages to initiate RIP3 signaling pathway, thus mediating macrophage/monocyte activation, and promoting the secretion of IL-6. Furthermore, the activation of TLR9 in immune cells by bacterial DNA activates NF-kB/MAPK signaling axis, and with it the secretion of IL-12 and TNF-α. Ah R ligand, derived from translocated *Enterococcus gallinarum*, activates AhR-CYP1A1 signaling pathway, thus inducing the production of Th17 cells and TFH cells. The decrease of secondary bile acids attenuates GPBAR1-IL10 axis, and with it, the decrease of NKT10 cells and IL10. PAMPs, pathogen associated molecular patterns; NLRs, NOD-like receptors; NLRP3, NOD-like receptor protein 3; IL-1β, interleukin-1β; LPS, lipopolysaccharide; TLR4/9, Toll-like receptor 4/9; MyD88, myeloid differentiation factor 88; IRAK4, IL-1R–associated kinase 4; NF-kB, nuclear factor kappa B; MAPK, mitogen-activated protein kinase; TRAM, TRIF-related adapter molecule; TRIF, TIR domain-containing adaptor inducing IFN-β; RIP3, receptor-interacting protein kinase 3; TNF-α, tumor necrosis factor-α; Ah R, aryl hydrocarbon receptor; CYP1A1, cytochrome P450 family 1A1; GPBAR1, G-protein coupled bile acid receptor 1; NKT cells, natural killer T cells; TFH, follicular helper T; HSC, hepatic stellate cell.

Intestinal microbiota and their metabolites are identified as great contributors to the occurrence and the development of AIH by activating multiple signaling pathways through binding to different receptors distributed in the liver and the intestine. Principal receptors implicated in the associated signaling pathways in the intestine consist of Toll-like receptor 4 (TLR4) (Guo et al., 2015) and G protein-coupled receptors (GPR41/GPR43, GPR109a) (Thangaraju et al., 2009; Kim et al., 2013; Zhao et al., 2018) (**Figure 1**), while in the liver, NLRs (Luan et al., 2018), TLRs (TLR4, TLR9) (Zhang et al., 2018; Liu et al., 2021a), Ah R (Manfredo Vieira et al., 2018) and GPBAR1 (Biagioli et al., 2019) take part in the progression of AIH (**Figure 2**).

#### In the Intestine

TLR4 is able to sense various exogenous and endogenous ligands, including LPS, hyaluronic acid, fatty acid and so on (Pradere et al., 2010). Recent studies have indicated that TLR4 plays a significant role in liver diseases in both humans and animals (Seki and Brenner, 2008; Pradere et al., 2010; Yang and Seki, 2012). The altered intestinal microbiota in AIH leads to the increase of LPS, which activates TLR4 expressed on IECs. The activated TLR4 results in the phosphorylation and activation of adaptor protein focal adhesion kinase (FAK) in IECs, which then modulates the activation of myeloid differentiation factor 88(MyD88) and IL-1R–associated kinase 4 (IRAK4), ultimately leading to intestinal barrier destruction and increased intestinal permeability (Guo et al., 2015). With a compromised intestinal barrier, gut-derived bacteria and metabolites are transferred from the intestine to mesenteric lymph nodes, systematic circulation, and the extraintestinal organs (Lin et al., 2015). When the gut-derived bacteria and their metabolites are translocated to the liver, they initiate a host of inflammation and immune responses, participating in the occurrence and progression of AIH (Luan et al., 2018; Manfredo Vieira et al., 2018; Zhang et al., 2018; Biagioli et al., 2019; Liu et al., 2021a).

GPRs are identified as the most diverse and largest membrane protein families, suggesting a conserved mechanism for extracellular signal perception in eukaryotic organisms (Pierce et al., 2002). SCFAs act as ligands for GPRs distributed on IECs to maintain intestinal barrier as well as immune homeostasis *via* the activation of a variety of signaling pathways in healthy conditions, including GPR41, GPR43 and GPR109a (Thangaraju et al., 2009; Kim et al., 2013; Zhao et al., 2018). To be more specific, the SCFA-dependent activation of GPR41/GPR43 distributed on IECs promotes cytokines and chemokines production by activating the p38 mitogen-activated protein kinase (MAPK) signaling pathway and the extracellular signal-related kinase (ERK)1/2 signaling pathway, which mediated protective immunity in mice (Kim et al., 2013). Moreover, by activating the signal transducers and activator of transcription 3 (STAT3), and mammalian target of rapamycin (mTOR) in a GPR43-dependent way, butyrate upregulates antimicrobial peptides (AMPs) secretion in IECs and regulates the interaction of intestinal bacteria (Zhao et al., 2018). Butyrate is also regarded as a critical mediator in anti-inflammatory responses, which inhibits nuclear factor kappa B (NF-kB) activation induced by LPS, and induces IL-18 secretion in IECs *via* the activation of GPR109a, thus protecting the intestine against inflammation (Thangaraju et al., 2009; Dupaul-Chicoine et al., 2010). Herein, the decrease of SCFAs caused by dysbiosis in AIH exacerbates the disruption of intestinal barrier and inflammation responses in the intestine, contributing to the translocation of gut-derived bacteria and their metabolites from the intestine to the liver.

In summary, the increment of LPS, induced by dysbiosis in AIH, activates the TLR4/FAK/MyD88 signaling pathway in IECs, leads to intestinal barrier destruction, and increases intestinal permeability (Guo et al., 2015). Similarly, the reduction of SCFAs in AIH exacerbates the intestinal barrier disruption and inflammatory injury *via* the inactivation of GPR41/43 and GPR109a expressed on IECs (Thangaraju et al., 2009; Kim et al., 2013; Zhao et al., 2018). The compromised intestinal barrier results in the translocation of gut-derived bacteria and intestinal metabolites from the intestine to the liver, where the translocated material activate multiple inflammation and immune responses, thus taking part in the initiation and progression of AIH (Luan et al., 2018; Manfredo Vieira et al., 2018; Zhang et al., 2018; Biagioli et al., 2019; Liu et al., 2021a).

#### In the Liver

Pattern recognition receptors (PRRs) in the liver can sense translocated pathogen associated molecular patterns (PAMPs), including NOD-like receptors (NLRs) and TLRs. NLRs have the capacity to sense various ligands within the cytoplasm. In general, NLRs can upregulate inflammatory cytokines secretion by activating MAPK, inflammasome or NF-kB by the recognition of PAMPs (Kumar et al., 2011). In an AIH mouse model induced by Con A, NOD-like receptor protein 3 (NLRP3) inflammasome activated by NLRs is identified as a critical mediator in the progression of Con A induced-hepatitis (Luan et al., 2018). To be more specific, the activated NLRP3 in macrophages contributes to the cleavage of caspase-1and the secretion of IL-1β, which then induces Th17 cells differentiation and recruits inflammatory cells. Therefore, the activated NLRP3 participates in various immune disease progression (Lasigliè et al., 2011; Pathak et al., 2011). Moreover, the secretion of IL-1β remarkably increased in AIH patients and was related to the aggravation of hepatitis in recent clinical studies (Longhi et al., 2012). To sum up, NLRs putatively exacerbate the progression of AIH mainly by activating NLRP3 inflammasome and its significant downstream molecules.

TLRs, the most extensively studied PRRs, recruit different adaptor molecules containing Toll/interleukin-1 receptor (TIR) domain, including TIR domain-containing adaptor inducing IFN-β (TRIF), TRIF-related adapter molecule (TRAM), and MyD88, to initiate different transcription factors including MAPK, IRF3/7 and NF-kB, thus inducing the secretion of proinflammatory cytokines (Kumar et al., 2011). In humans, ten TLR family members are identified, whereas there are twelve identified TLR family members that are extensively distributed in different cells in mice (Kumar et al., 2011). Dysbiosis in AIH patients leads to increment of PAMPs in the liver, such as LPS and bacterial DNA, which can bind to TLRs distributed in the liver and initiate a series of signaling pathways. Specifically, in hepatic stellate cells (HSCs), Kupffer cells, and hepatocytes, the activation of TLR4 by LPS contributes to proinflammatory chemokines and cytokines secretion by activating MAPK, IRF3, and NF-KB, which results in hepatic injury and fibrotic progression in AIH (Liu et al., 2021a). Moreover, the activation of TLR4 on macrophages initiates receptor-interacting protein kinase3 (RIP3) signaling pathway, thus mediating the activation of macrophage/monocyte in the liver, and promoting the secretion of IL-6 (Zhang et al., 2018). IL-6 can not only stimulate B cells to drive the production of IgG (Kishimoto, 1989), but also switch Tregs to Th17 cells in other autoimmune diseases (Bettelli et al., 2006; Mangan et al., 2006; Veldhoen et al., 2006). Therefore, it appears plausible to speculate that IL-6 contributes significantly to the initiation and the progression of AIH. In addition, the activated TLR4/MyD88 signaling pathway in immune cells induced by LPS also contributes to the imbalance of follicular regulatory T (TFR)/follicular helper T (TFH) cells (Levy et al., 2017). Such imbalance ultimately results in immune homeostasis breakdown and excessive autoantibodies production (Liang et al., 2020), thus playing a significant role in the pathogenesis of AIH (Liang et al., 2021). Furthermore, the activation of TLR9 by bacterial DNA initiates the NF-kB/MAPK signaling pathway in immune cells, and with it the secretion of IL-12 and TNF-α, which can exacerbate hepatic inflammatory injury in AIH (Liu et al., 2021a).

The aryl hydrocarbon receptor (Ah R) belongs to one kind of ligand-activated transcription factors and the basic region-helix-loop-helix (bHLH) superfamily of DNA binding proteins (Burbach et al., 1992), which is extensively distributed at barrier sites, including the skin, lung, gut and so on (Metidji et al., 2018). Substantial low-molecular-weight substances, such as tryptophan metabolites and indoles, act as ligands for Ah R (Denison and Nagy, 2003; Nguyen and Bradfield, 2008). Upon the recognition of Ah R ligands, Ah R is translocated from cytoplasmic to nucleus where it induces the transcription of target genes with the promoter. The promoter contains the xenobiotic-response element (XRE) sequence after dimerization with Ah R nuclear translocator (ARNT), including Ah R-cytochrome P450 family 1A1 (CYP1A1) (Sogawa and Fujii-Kuriyama, 1997). *Enterococcus gallinarum*, which is translocated to the liver from the intestine induced by disrupted intestinal barrier in AIH, encodes shikimic acid pathway, produces Ah R ligand, and activates AhR-CYP1A1 signaling pathway, ultimately promoting the transcription of CYP1A1 (Manfredo Vieira et al., 2018). The activated AhR-CYP1A1 pathway induces the production of TFH cells and Th17 cells (Veldhoen et al., 2008; Moura-Alves et al., 2014; Schiering et al., 2017), which is conducive to the secretion of systematic autoantibodies, thus putatively participating in the initiation of AIH (Manfredo Vieira et al., 2018).

GPBAR1 belongs to one kind of GPRs, which is extensively distributed in hepatic nonparenchymal cells, including cholangiocytes, activated HSCs, sinusoidal endothelial cells, as well as Kupffer cells (Keitel and Häussinger, 2012; Sawitza et al., 2015). Secondary bile acids are identified as ligands for GPBAR1 (Maruyama et al., 2002; Kawamata et al., 2003). In a recent study, GPBAR1-IL10 axis was reported to serve as a great contributor to the progression of Con A-induced hepatitis in a mouse model (Biagioli et al., 2019). To be more specific, GPBAR1 modulates the differentiation of type I and type II NKT cells in hepatic immune systems, and polarizes NKT cells to NKT 10 cells, which stimulates the secretion of anti-inflammatory cytokines IL10, thereby remarkably mitigating immune-mediated hepatitis induced by Con A (Biagioli et al., 2019). Therefore, the decrease of secondary bile acids owing to the reduced abundance of *Colstridium* in AIH (Kayama et al., 2020), exacerbates hepatic inflammatory injury *via* the inhibition of GPBAR1-IL10 axis.

In summary, dysbiosis in AIH results in the translocation of intestinal bacteria together with their products from the intestine to liver. NLRs recognize translocated PAMPs in the liver, which enhances the secretion of proinflammatory cytokines, thus aggravating hepatic inflammatory injury in AIH (Kumar et al., 2011; Luan et al., 2018). TLRs, specifically TLR4 and TLR9, are activated by LPS and bacterial DNA, respectively. The activated TLRs contribute to the breakdown of hepatic immune homeostasis, the excessive secretion of autoantibodies, as well as the production of proinflammatory chemokines and cytokines, thereby participating in the occurrence and development of AIH (Zhang et al., 2018; Liu et al., 2021a). Moreover, translocated *Enterococcus gallinarum* in the liver activates AhR-CYP1A1 signaling pathway to promote the production of systematic autoantibodies, which is likely to take part in the initiation of the disease (Manfredo Vieira et al., 2018). Additionally, the decrease of secondary bile acids represses GPBAR1-IL10 axis, thus aggravating hepatic inflammatory injury in AIH (Biagioli et al., 2019). Herein, the afore-mentioned signaling pathways activated by different receptors distributed in the liver make great contributions to the onset and progression of AIH.

### Immune Pathway

As a chronic immune-mediated inflammatory liver disorder, great significance should be attached to unravel the critical role of immune responses in AIH. Some studies have suggested that the dysregulation between Tregs and Th17 cells (Liu et al., 2021b), the activation of NKT cells (Diao et al., 2004; Liu et al., 2021a), and the imbalance of TFR/TFH cells induced by altered intestinal microbiota, presumably participated in the initiation and the progression of AIH (Liang et al., 2021).

The alterations of intestinal microbiota in AIH patients reduce the proportion of Tregs as well as raise the proportion of Th17 cells through exerting influence on the metabolism of luminal contents (Mizuno et al., 2017; Lou et al., 2020). Th17 cells promote proinflammatory cytokines secretion, including TNF-α, IL-22 and so on, which aggravates immune attack and inflammatory injury in the liver (de Oliveira et al., 2017). In contrast, Tregs release TGF-β and IL10 to repress the immune effector cells’ activation, or restrain their function by interacting with dendritic cells (DCs), thereby regulating immune homeostasis (Chen et al., 2019; Wang et al., 2020). Herein, the increased Th17 cells and decreased Tregs induced by altered intestinal microbiota in AIH disrupt immune homeostasis and exacerbate inflammatory injury, putatively contributing to the progression of the disease. Moreover, the evidence that the Treg/Th17 cells ratio had an intimate association with the disease severity (Liu et al., 2021b) further supported the linkage between imbalanced Treg/Th17 cells and the progression of AIH.

In Con A-induced fulminant hepatitis, a condition similar to AIH patients, NKT cells in the liver can be activated by intestinal pathogens through two pathways. One pathway may be that intestinal pathogens initiate the activation of intestinal DCs. The intestinal DCs then migrate to the liver through Peyer patches (PPs), contributing to the activation of hepatic NKT cells. The other pathway is likely to be that a great host of translocated intestinal antigens first move to the liver, activate liver DCs, and subsequently activate NKT cells (Chen et al., 2014). The activation of NKT cells further activates Kupffer cells and recruits macrophages to secrete numerous inflammatory cytokines, which initiates the repairing responses including hepatocyte regeneration as well as fibrosis through activated HSCs (Diao et al., 2004; Liu et al., 2021a). Together, they contribute to the aggravation of hepatic inflammatory injury and fibrotic progression in AIH.

The elevated LPS in AIH disease model inhibited TFR cells and activated TFH cells by activating TLR4/MyD88 signaling pathway (Levy et al., 2017). The excessively activated TFH cells are intimately related to hypergammaglobulinemia, which accelerates the immunopathological process of AIH (Liang et al., 2020). TFR cell indirectly inhibits the activation of TFH cells upon the recognition of the coreceptor CLTA4, thus reducing the production of autoantibody (Liang et al., 2021). Therefore, the imbalance of TFR/TFH cells led to the destruction of immune homeostasis and the excess autoantibodies secretion, therefore taking part in the immunopathological process in AIH (Liang et al., 2021). Moreover, in a recent study, the dysregulation between TFH and TFR cells was augmented in EAH model mice after administration with broad-spectrum antibiotics (Liang et al., 2021), further supporting the intimate linkage between imbalanced TFR/TFH cells and AIH immunopathological process.

In summary, dysregulation between Tregs and Th17 cells leads to proinflammatory cytokines secretion and immune responses aggravation, and contributes to the progression of AIH (Liu et al., 2021b). The activated NKT cells are conducive to initiating repairing responses in the liver, as well as the production of proinflammatory cytokines (Diao et al., 2004; Chen et al., 2014; Liu et al., 2021a), which aggravate hepatic inflammatory injury and fibrotic progression in AIH. Moreover, the imbalance of TFR/TFH cells accelerates the pathological process of AIH by upregulating the secretion of autoantibodies (Liang et al., 2020; Liang et al., 2021). Therefore, the afore-mentioned alterations of immune cells or responses induced by dysbiosis in AIH give impetus to the progression of the disease.

## Novel Methods Targeting for Microbiota to Attenuate AIH

Currently, the primary therapeutic methods for AIH are glucocorticoid or a combination with azathioprine, which effectively alleviate symptoms and prolong life in the majority of patients with AIH (Mack et al., 2020). However, some patients are still not tolerating standard management or not achieving remission. In addition, some detrimental effects of glucocorticoid and azathioprine cannot be ignored, such as central obesity, osteoporosis, myelosuppression, and liver function damage (Lee et al., 2014; Mack et al., 2020). Given that altered intestinal microbiota contributes greatly to the onset and progression of AIH, restoring intestinal microbiota putatively represents a new revenue for treating AIH. To date, probiotics (Lou et al., 2020; Zhang et al., 2020; Liu et al., 2021b), fecal microbiota transplantation (FMT) (Liang et al., 2021; Wang et al., 2021a), and some pharmacological agents targeted intestinal microbiota-associated signaling pathways (Frasca et al., 2012; Scaldaferri et al., 2014; Telesford et al., 2015; Hsu et al., 2017) have been confirmed to attenuate autoimmune hepatitis in AIH model mice, which putatively constitute a promising therapy for patients with AIH.

### Probiotics and its Therapeutic Mechanisms

#### Probiotics

The international society of probiotics and prebiotics defines probiotics as “living microorganisms which are conducive to the health state of the host if administered in sufficient quantities” (Beyaz Coşkun and Sağdiçoğlu Celep, 2021). Currently, the most frequently used species are *Lactobacillus* and *Bifidobacterium*, and they have been evaluated as a useful therapy for the prevention or treatment of gastrointestinal infections, urogenital infections, periodontal diseases, as well as dental caries (Caglar et al., 2005; White et al., 2017; Scorletti et al., 2020). In addition, probiotics are also conducive to treating immune-mediated diseases by regulating systematic immune responses (Vleggaar et al., 2008; De Filippis et al., 2021; Wang et al., 2022).

Liu et al. (2021b) have administered compound probiotics through gavage and dexamethasone through intraperitoneal injection to the AIH model mice for 42 days. The result suggested that the afore-mentioned interventions ameliorated liver inflammatory responses, and decreased the level of Th1 and Th17 cells, and serum aminotransferase. In addition, Tregs increased just in the probiotic group, indicating that compound probiotics have immunomodulatory effects. Zhang et al. (2020) found that B420 could revert the altered intestinal microbiota in the EAH model mice induced by S100 to normal, strengthen the function of the intestinal barrier, and alleviate inflammatory injury in the liver, thereby remarkably mitigating EAH induced by S100. Lou et al. (2020) illustrated that *Ruminococcus* decreased the frequency of Th1 and Th17 cells, inhibited the activation of effector T cells, and induced IL10 expression, further modulating intestinal homeostasis. These studies indicated that probiotics offered the promise of novel therapy in AIH.

#### The Associated Mechanisms of Probiotics in the Treatment of AIH

Probiotics make contributions to the treatment of AIH *via* multiple mechanisms. Firstly, probiotics compete with pathogenic bacteria for necessary nutrients and common adhesion receptors, thus affecting their survival and colonization (Bron et al., 2017). Herein, compound probiotic treatment is of great benefit to reduce harmful bacteria abundance and increase beneficial bacteria abundance in the intestine (Liu et al., 2021b). Secondly, microbiome related molecular patterns (MAMPs) from probiotics can activate PRRs expressed on the intestinal mucosa, thus enhancing intestinal barrier function mainly by upregulating the synthesis of tight junction proteins and enhancing their function (Bron et al., 2017). The integral intestinal barrier blocks the translocation of gut-derived pathogenic microorganisms as well as their metabolites. This blockage can inhibit the RIP3 signaling pathway in liver macrophages (Zhang et al., 2020), and repress TLR4/NF-kB signaling pathway in the liver and the intestine (Liu et al., 2021b), which conspicuously alleviates hepatitis induced by immune factors.

In addition, probiotics such as *Lactobacillus* can promote the production of SCFAs (Zhang et al., 2020), which are capable of triggering a variety of signaling pathways to modulate intestinal barrier function as well as immune homeostasis by binding to GPR41/43 and GPR109a (Thangaraju et al., 2009; Kim et al., 2013; Zhao et al., 2018). Besides SCFAs, lactic acid from probiotics also makes contributions to maintain intestinal barrier integrity. On the one hand, lactate induces Wnt3 expression in Paneth cells and stromal cells by binding to the lactate specific receptor GPR81, enhancing the proliferation of epithelial stem cells, thus preventing intestinal damage. On the other hand, lactic acid modulates immune responses by affecting CX3CR1+ phagocytes in the lamina propria, which enter the lumen to absorb luminal harmful bacteria by expanding dendrites (Kayama et al., 2020). Furthermore, the activation of NF-kB (MyD88) *via* TLR signaling pathway by probiotics triggers the expression of antimicrobial factors and intestinal epithelial defensins in Paneth cells, thus promoting the production of AMPs (Bron et al., 2017), and inhibiting the survival and colonization of intestinal pathobiont. In conclusion, probiotics can ameliorate the adverse condition of AIH by regulating intestinal microbiota composition and maintaining intestinal barrier and immune homeostasis.

### Fecal Microbiota Transplantation

An introduction of the functional microbiota from the feces of healthy donors into the gut tract of patients is termed FMT (Milosevic et al., 2019), which aims at the reconstruction of new intestinal microbiota, and to exhibit potential efficacy against gastrointestinal and extra-gastrointestinal diseases (Beyaz Coşkun and Sağdiçoğlu Celep, 2021). Owing to the successful *C. difficile* fecal transplantation, more and more patients have registered for fecal transplantation, especially those with gastrointestinal diseases such as metabolic syndrome or inflammatory bowel disease (IBD) (Bron et al., 2017). Indeed, some studies with regard to metabolic syndrome (Vrieze et al., 2012) and ulcerative colitis (UC) (Borody et al., 2003) delineated that FMT ameliorated insulin resistance or prolonged the length of remission period, respectively, shedding light on the potential of FMT to treat microbiota-related diseases. Furthermore, some studies indicated that FMT had the capacity to effectively ameliorate hepatitis in EAH model mice putatively by restoring the composition of intestinal microbiota and rectifying the imbalance of TFR/TFH cells (Liang et al., 2021). Moreover, in a recent study, the antibiotic-treated mice exhibited AIH phenotypes after being transplanted with fecal microbiota from mice exposed to TCE, concomitantly with increased systematic autoantibodies and aggravated hepatic inflammation compared to the controls (Wang et al., 2021a). The afore-mentioned evidence verified the underlying therapeutic function of FMT in immune-mediated diseases.

### Pharmacological Agents Targeted Intestinal Microbiota-Associated Pathways

#### Gelatin Tannate

The integrity of intestinal barrier is destructed by high dose LPS induced by the alterations of intestinal microbiota in AIH, and the resulting increased intestinal permeability (Guo et al., 2015; Lin et al., 2015). Restoring the compromised intestinal barrier blocks the translocation of intestinal bacteria and their metabolites from the gut to the liver (Lopetuso et al., 2015), thereby attenuating hepatic injury and fibrotic progression. Gelatin tannate constitutes a “mucus-like” shield for compromised intestinal mucosa, promoting the intestinal mucosal healing process and reducing intestinal leakage. The decreased blood LPS level in groups treated with gelatin tannate compared to controls further confirms its ability to restore shield activity of mucus layer (Scaldaferri et al., 2014). Moreover, gelatin tannate shows potent anti-inflammatory properties by inhibiting inflammatory biomarkers, such as TNF-α, IL-8, and intercellular adhesion molecule-1 (ICAM-1) in human epithelial colorectal adenocarcinoma (Caco-2) cells (Frasca et al., 2012).

#### JKB-122

TLR4, a significant cell surface receptor, takes part in the progression of AIH through activating multiple intracellular signaling pathways, which further bring about intestinal barrier destruction (Guo et al., 2015), promoting the production of proinflammatory cytokines (Liu et al., 2021a), and disrupting immune homeostasis (Zhang et al., 2018). JKB-122, as a TLR4 antagonist, conferred protection against Con A-induced hepatitis in mice, and exhibited anti-inflammatory properties mainly through suppressing proinflammatory cytokines production in both serum and liver, such as TNF-α, interferon γ (IFN-γ), IL5, IL6, as well as IL17 in a dose-dependent way (Hsu et al., 2017). Moreover, in a translational model of AIH, JKB-122 was proved to be effective alone or with prednisolone, which requires rigorous evaluation and further investigation (Hsu et al., 2017).

#### Polysaccharide A

The imbalance of Th17/Treg cells significantly contributes to the development of AIH (Liu et al., 2021b). Polysaccharide A, as a symbiosis factor derived from human commensal *Bacteroides fragilis*, could promote the immunologic development of mammalian hosts. It has been reported to induce Foxp3^+^ Tregs production in mice, which inhibited the activity of Th17 cells, thereby rectifying imbalanced Th17/Treg cells (Telesford et al., 2015). Therefore, it is reasonable to speculate polysaccharide A offers the promise of a novel therapy for patients with AIH.

#### BAR 501

GPBAR1-IL10 axis has been confirmed to make contributions to the progression of AIH (Biagioli et al., 2019). BAR 501, as a potent agonist of GPBAR1, polarized NKT cells to NKT10 cells, and enhanced IL-10 secretion, which almost completely reversed inflammatory injury in the liver induced by Con A at a dose of 30-mg/kg in some preliminary experiments (Biagioli et al., 2019). Herein, BAR 501 putatively represents a novel therapy for patients with AIH.

## Conclusion

AIH is a chronic immune-mediated inflammatory liver disease with obscure etiology (Cowling et al., 1956; Manns et al., 2010). An accumulating body of evidence highlights the importance of “intestinal liver crosstalk” in AIH pathogenesis (Lin et al., 2015). Many studies delineated the alterations of the intestinal microbiome in AIH disease model (Lin et al., 2015; Yuksel et al., 2015; Wei et al., 2020). The transformation of intestinal microbiota from anaerobic to aerobic (Wei et al., 2020) altered the immune responses and the metabolism of luminal contents (Lin et al., 2015), including the imbalance of Treg/Th17 cells and TFR/TFH cells, the activation of NKT cells, the increase of PAMPs, and the decrease of SCFAs and secondary bile acids, which subsequently lead to the destruction of the intestinal barrier, breakdown of immune homeostasis, augmentation of inflammatory injury and progression of fibrosis *via* multiple mechanisms, including receptor, immune and metabolites pathway. For patients not tolerating standard management or not achieving remission, probiotics, FMT, as well as some pharmacological agents targeted intestinal microbiota-associated pathways seem to represent new avenues for treatments for patients with AIH by restoring intestinal microbiota composition and modulating immune responses.

However, some knowledge gaps concerning AIH and intestinal microbiota still exist. For instance, predominant analyses of the composition and diversity of intestinal microbiota mainly depend on fecal samples from patients with AIH or AIH animal models, which are not able to completely reflect the abundance and composition of mucosal communities. Therefore, there is an urgent need to pay more attention to investigate mucosal microbiota, which is conducive to clarifying the alterations of intestinal microbiota in AIH. In addition, some studies have controversial results concerning the alterations of some specific species of intestinal microbiota, which warrant further investigation. Furthermore, with the complexity of microbial communities, the safety of FMT cannot be fully evaluated. Lack of evidence from clinical patients and a high rate of misdiagnosis and missed diagnosis in clinical practice are also problems to be solved. Herein, large scale patient follow-up and controlled prospective studies are still warranted to unravel the relationship between AIH and intestinal microbiota. More understanding regarding this relationship could provide direct evidence for the underlying mechanism of intestinal microbiota in AIH, offer favorable guidance for the treatment which targets intestinal microbiota, and supply the theoretical basis for the formulation of diagnosis and treatment guidelines for AIH.

## Author Contributions

ZC collected the literatures and drafted the manuscript. LY and HC critically revised the manuscript. All authors read and approved the final version of the manuscript.

## Funding

This study was supported by the National Natural Science Foundation of China (No.82000561 to HC; No.81974078,81570530, 81370550 to LY), Natural Science Foundation of Hubei Province (No.2019ACA1333 to LY) and the Science foundation of union hospital (No. 2021xhyn005 to HC).

## Conflict of Interest

The authors declare that the research was conducted in the absence of any commercial or financial relationships that could be construed as a potential conflict of interest.

## Publisher’s Note

All claims expressed in this article are solely those of the authors and do not necessarily represent those of their affiliated organizations, or those of the publisher, the editors and the reviewers. Any product that may be evaluated in this article, or claim that may be made by its manufacturer, is not guaranteed or endorsed by the publisher.

## Abbreviations

AIH, autoimmune hepatitis; FMT, fecal microbiota transplantation; Con A, concanavalin A; TCE, trichloroethene; AUC, area under curve; AST, aspartate aminotransferase; LPS, lipopolysaccharide; ALD, alcohol-associated liver disease; NAFLD, non-alcohol fatty liver disease; SCFA, short-chain fatty acid; B420, *Bifidobacterium* animal lactic acid 420; EAH, experimental autoimmune hepatitis; IECs, intestinal epithelial cells; TNF-α, tumor necrosis factor-α; IL, interleukin; GPBAR1, G-protein coupled bile acid receptor 1; NKT, natural killer T; GPR, G protein-coupled receptor; TLR, Toll-like receptor; FAK, adaptor protein focal adhesion kinase; MyD88, myeloid differentiation factor 88; IRAK4, IL-1R–associated kinase 4; ERK, extracellular signal-related kinase; MAPK, mitogen-activated protein kinase; AMPs, antimicrobial peptides; STAT3, signal transducers and activator of transcription 3; mTOR, mammalian target of rapamycin; NF-kB, nuclear factor kappa B; PPRs, pattern recognition receptors; PAMPs, pathogen associated molecular patterns; NLRs, NOD-like receptors; NLRP3, NOD-like receptor protein 3; TIR, Toll/interleukin-1 receptor; TRIF, TIR domain-containing adaptor inducing IFN-β; TRAM, TRIF-related adapter molecule; HSCs, hepatic stellate cells; TFR, follicular regulatory T; TFH, follicular helper T; Ah R, aryl hydrocarbon receptor; bHLH, basic region-helix-loop-helix; XRE, xenobiotic-response element; ARNT, Ah R nuclear translocator; CYP1A1, cytochrome P450 family 1A1; DCs, dendritic cells; PPs, Peyer patches; MAMPs, microbiome related molecular patterns; IBD, inflammatory bowel disease; UC, ulcerative colitis; ICAM1, intercellular adhesion molecule-1; Caco-2, colorectal adenocarcinoma; IFN-γ, interferon γ.
